# Association of Subclinical Hypothyroidism With Anxiety Symptom in Young First-Episode and Drug-Naïve Patients With Major Depressive Disorder

**DOI:** 10.3389/fpsyt.2022.920723

**Published:** 2022-06-24

**Authors:** Ruchang Yang, Xiangdong Du, Zhe Li, Xueli Zhao, Xiaoli Lyu, Gang Ye, Xinchuan Lu, Guangya Zhang, Chuanwei Li, Yan Yue, Yuxuan Wu, Ruijie Peng, Yue Zhou, Haitao Wang, Siqi Wu, Pallavi B. Ganapathi, Hanjing Emily Wu, Xiangyang Zhang

**Affiliations:** ^1^Suzhou Medical College of Soochow University, Suzhou, China; ^2^Suzhou Guangji Hospital, The Affiliated Guangji Hospital of Soochow University, Suzhou, China; ^3^Xuzhou Medical University, Xuzhou, China; ^4^School Psychology and Mental Health, North China University of Science and Technology, Tangshan, China; ^5^Department of Psychiatry and Behavioral Sciences, The University of Texas Health Science Center at Houston, Houston, TX, United States; ^6^CAS Key Laboratory of Mental Health, Institute of Psychology, Chinese Academy of Sciences, Beijing, China

**Keywords:** subclinical hypothyroidism, anxiety symptom, young, major depressive disorder, first-episode and drug-naïve

## Abstract

**Backgrounds:**

Subclinical hypothyroidism (SCH) was reported to be associated with depression; however, its role in coexisting anxiety symptom in young patients with major depressive disorder (MDD) remains unclear. The objective of this study was to explore the relationship between SCH and anxiety symptom in young first-episode and drug-naïve (FEDN) MDD patients.

**Methods:**

A total of 520 outpatients diagnosed as FEDN MDD with SCH were recruited in this study. Their socio-demographic, clinical data and thyroid function parameters were collected. The Hamilton Anxiety Rating Scale (HAMA) and the Hamilton Depression Rating Scale (HAMD) were employed to measure the severity of anxiety symptom and depressive symptom, respectively. Based on the HAMA scores, patients who scored ≥ 25 were defined as anxious major depressive disorder (A-MDD) while others as non-anxious major depressive disorder (NA-MDD).

**Results:**

The prevalence rate of A-MDD was 15.8% in young FEDN MDD patients with comorbid SCH. Moreover, serum thyroid stimulating hormone (TSH) levels were significantly higher in patients with A-MDD compared with those with NA-MDD (*p* < 0.001). Multivariate binary logistic regression analysis indicated that A-MDD was associated with serum TSH levels with an odds ratio (OR) of 1.602. Serum TSH level of 6.17 mIU/L was the critical value to distinguish A-MDD and NA-MDD, with sensitivity of 0.805 and specificity of 0.539. There were no statistically significant differences between NA-MDD and A-MDD patients in terms of socio-demographic variables, serum free triiodothyronine (FT3), free thyroxine (FT4), thyroid peroxidases antibody (TPOAb) and anti-thyroglobulin (TgAb) levels.

**Conclusions:**

A-MDD patients presented higher serum TSH level. It is suggested that serum TSH level may be a potential biomarker for predicting moderate and severe anxiety symptoms in young FEDN MDD patients with SCH.

## Introduction

Major depressive disorder (MDD) is the most prevalent mood disorder in China, with a lifetime prevalence of 3.4% and 12-month prevalence of 2.1% (1). MDD is a common reason for years lived with disability with a high incidence rate, causing great burden to individuals and society (2, 3). Previous studies suggest young adults are at serious risk of MDD. A multinational study showed 12-month prevalence estimates of MDD between 8.3 and 12.4% among people aged 18–33 (4), while the overall 12-month prevalence of MDD is approximately 6% (5). A large sample study in Singapore reported a higher risk for adults aged 18–34 than other age groups to suffer from MDD (6).

Anxiety is one of the most commonly seen comorbidities in MDD patients, with a prevalence of 45–67% (7). Patients with anxious depression, which has been characterized as MDD with high levels of anxiety symptoms, were more likely to experience serious psychiatric symptoms, more severe depression, and a greater risk of suicide (8). In addition to psychological impairments, physical comorbidities such as pain, hypertension and diabetes mellitus are particularly elevated among patients with co-occurring depressive and anxiety disorders (9).

There is a considerably strong correlation between mood disorders and thyroid function (10, 11). Thyroid hormones (TH) are critical not only for brain development, but also for lifelong central nervous system (CNS) function (11–13). Numerous studies have demonstrated that TH affect depression and anxiety through multiple mechanisms. For example, Buras et al. noted that TH act via the thyroid hormone receptor (TR) α and β isoforms. Both isoforms are expressed in the limbic system, which plays an important role in mood regulation (14). Yu et al. demonstrated that by modulating hippocampal brain-derived neurotrophic factor (BDNF) level, thyroid dysfunction has bidirectional effects on anxiety- and depression-like behaviors (15). TH may also modulate the brain serotonergic system (16), further influencing anxiety and depression.

Anxiety and depression are highly prevalent among patients with hypothyroidism (11). Multiple studies have focused on the role of thyroid function in depression and anxiety, but no consistent conclusion has been reached. Erensoy recommended serum thyroid stimulating hormone (TSH) as a useful biochemical marker for more efficient depression management (17), while a systematic review on hypothalamic-pituitary-thyroid (HPT) axis function in anxiety disorders implicated a negative relationship between self-reported anxiety and TSH levels (18). This may result from different inclusion criteria such as age, gender, also suggesting further investigation. Yet Yu and colleagues suggested a bidirectional effect of thyroid function toward anxiety and depression (15). A retrospective study in China suggested thyroid hormones can reflect the clinical outcome of depression and normal-range free thyroxine (FT4) values are associated with the severity of anxiety and depression (12). Ittermann et al. found serum thyroid peroxidases antibody (TPOAb) levels to share no apparent correlation with depression and anxiety (19), while van de Van and colleagues suggested TPOAb to be a predictive biomarker for the vulnerability of depression (20).

With the increasingly precise testing and widespread screening during routine physical examination, subtle degrees of thyroid dysfunction can be discovered at the subclinical stage and diagnosed in young and middle-aged people at a higher frequency than ever before. Subclinical thyroid disease (SCTD) is defined as normal serum FT4 and free triiodothyronine (FT3) levels in the presence of abnormal serum TSH levels (44), high and low abnormality for subclinical hyperthyroidism and subclinical hypothyroidism (SCH), respectively (44). However, there are insufficient studies on the association between SCH and anxiety and depression, and no unanimous consensus has been reached. A systematic review suggested a higher risk of depression and anxiety in SCH patients (21), implying the significant role thyroid may play in regulating mood disorders. Zhao et al. found that recurrent and high body mass index (BMI) female depressed inpatients to be at increased risk of developing SCH. Stress likely plays a crucial role here, as depressed females are more vulnerable to stressors, leading to increased TSH level, thyroid dysfunction, psychological problems, and overeating (22). However, Bensenor et al. found SCH was negatively associated with anxiety disorder, but the result lost statistical significance after adjustment for multiple comparisons (23). A large sample study in China suggested depression and anxiety symptoms are correlated with severe SCH and elevated TSH levels (24).

Association between thyroid function and depression and anxiety can differ significantly for age, races and different disease episodes. A cross-sectional study in Europe suggested hypothyroidism is relevant with the severity of depression and its psychopathologic features in older people of Caucasian origin (2). Similarly, a Western Pomerania population-based study concluded that diagnosed untreated hypothyroidism is associated with depression and anxiety (19). Apart from races, the endocrine system changes in different life periods. For instance, there is a drastic difference before and after climacteric, typically in middle-aged people. It should be noted that endogenous depression or menopause-relative psychological manifestation may affect the assessment of psychiatric disorders (11). Moreover, it is considered that the titer of antibodies, such as TPOAb may vary with the development of disease (25). All these factors above may render inconsistent results of thyroid function in depression and anxiety, particularly because participants' age, race and physical status are not preserved across different studies.

Though many previous studies have demonstrated the relevant association between hypothyroidism and anxiety and depression, few studies focused on the potential association between SCH and anxiety and depression. By exploring at the subclinical stage, we can further understand the role of the thyroid and HPT axis in the occurrence and development of A-MDD. This may contribute to greater comprehension regarding pathogeny and early intervention. Moreover, with the introduction of TH therapy to depression treatment in recent years (26, 27), it may also provide evidence and bolster support for clinical practice. SCH has a different diagnostic standard and hormone level than clinical hypothyroidism; thus, results drawn from clinical hypothyroidism patients should be extended to SCH with caution. Even within SCH patients, ordinary thyroid indicators such as TSH and TPOAb may vary with age and race. Medication (15) or other antidepressant treatments (28) could also affect physical hormone levels. Therefore, our study recruited 520 Chinese Han population with first-episode and drug-naïve major depressive disorder (FEDN MDD), where the influence of antidepressant treatment on thyroid relevant indicators can be ignored, to explore the associations of SCH with anxiety. To minimize disturbances caused by aging, all the participants are in the age group of 18–35. To our best knowledge, no prior study has focused on this specific population to explore the association between SCH and anxiety symptom. The purposes of this study were to: (1) examine the relationship between SCH and anxiety in young FEDN MDD patients; (2) identify contributors that are significantly associated with anxiety symptom in young FEDN MDD with SCH.

## Methods

### Subjects

From 2015 to 2017 this cross-sectional study included 520 outpatients from a psychiatric clinic at a tertiary general hospital in China.

The study inclusion criteria were: (1) Han nationality; (2) age between 18 and 35 years; (3) a diagnosis of MDD according to DSM-IV; (4) first episode patients with no previous medication history; (5) course of illness ≤24 months; (6) a score of ≥24 on the 17-item Hamilton Rating Scale for Depression (HAMD-17); (7) serum TSH level>4.20 mIU/L (upper limit of normal value), serum FT3 and FT4 level within their respective reference ranges; (8) no previous thyroxine therapy, or any specific medications. Exclusion criteria were: (1) having a serious physical disease; (2) pregnancy or lactation; (3) alcohol or substance dependence or abuse except for tobacco smoking.

All participants were voluntarily recruited and signed a written informed consent before enrollment. The medical ethics committee in First Hospital of Shanxi Medical University approved this study.

### Socio-Demographic Characteristics

Socio-demographic characteristics including age, gender, age at onset, illness duration, marital status, education level and BMI were collected by well-trained researchers.

### Clinical Measures

In this study, the severity of depression was assessed by the 17-item Hamilton Depression Scale (HAMD) (29). The 14-item Hamilton Anxiety Rating Scale (HAMA) was employed to measure anxiety severity (30). Each item in HAMA was scored on a scale of 0–4, with a total score of 56 points. Participants who scored 0–17 were considered as having no anxiety, 18–24 as mild anxiety, 25–29 as moderate anxiety, and ≥ 30 as severe anxiety (31). Based on the HAMA scores, we defined patients who scored ≥ 25 as anxious major depressive disorder (A-MDD) while others as non-anxious major depressive disorder (NA-MDD) (32). They were distributed to A-MDD and NA-MDD subgroup, respectively, and compared socio-demographic and clinical data between two groups.

The information above was collected by two qualified psychiatrists with no prior knowledge of participants' clinical conditions. After repetitive evaluation, the inter observer correlation coefficients of the HAMD and HAMA total score were both >0.8.

### Blood Sample

Participants' blood samples were taken between 6:00 and 8:00 a.m. after an overnight fast, and sent for testing before 11 a.m. The laboratory center of the hospital was responsible for the measurement of serum levels of FT3, FT4, TSH, anti-thyroglobulin (TgAb), and TPOAb, with Roche C6000 Electrochemiluminescence Immunoassay Analyzer (Roche Diagnostics, Indianapolis, IN, USA). The normal range was 0.27–4.20 mIU/L for TSH, 3.10–6.80 pmol/L for FT3, 10–23 pmol/L for FT4, 0–115 IU/L for TgAb and 0–34 IU/L for TPOAb.

### Statistical Analysis

The Kolmogorov-Smirnov test was used to assess the normality of all variables. For those variables that were normally distributed, they were expressed as mean ± standard deviation (SD), and parametric tests were performed and analysis of variance (ANOVA) was used for group comparisons. For those variables that were not normally distributed, they were expressed as median [interquartile range, IQR] and a non-parametric Mann-Whitney U test was performed to compare differences between groups. For those categorical variables, they were expressed as absolute numbers (percentages) and a chi-square test was performed to compare group difference. A univariate binary logistic regression was performed, in which A-MDD was used as dependent variable and serum TSH level was used as independent variable. After controlling for demographic and clinical characteristics, a multivariate binary logistic regression analysis was performed, in which A-MDD was used as dependent variable and age, illness duration, age at onset, gender, educational level, marital status, BMI, HAMD, TSH, TgAb, TPOAb, FT3, and FT4 were used as independent variables, to assess the factors associated with A-MDD. Receiver-operating characteristic (ROC) curve was constructed to assess the diagnostic accuracy of serum TSH level. Statistical analysis was computed using SPSS 23 with two-tailed *p*-values of 0.05.

## Results

### Socio-Demographic and Clinical Characteristics

As shown in Table 1, a total of 520 participants met the inclusion criteria. Among them 82 (15.8%) met the criteria for A-MDD and 438 (84.2%) were NA-MDD. Compared with NA-MDD, there were no significant differences in age, illness duration, age at onset, gender, HAMD score, serum TgAb, TPOAb, FT3, and FT4 levels and BMI. The only significant difference between the groups was in serum TSH level (*p* < 0.001), with the A-MDD subgroup displaying a higher level than the NA-MDD subgroup.

**Table 1:** Socio-demographical and clinical characteristics of the participants.

**Variable**	**Young FEDN MDD patients with SCH**		***χ^2^/z/F***	***P***
	**NA-MDD (*N* = 438)**	**A-MDD (*N* = 82)**		
**Socio-demographic and clinical characteristics**				
Age, mean ± SD, y	24.81 ± 5.36	24.49 ± 5.26	0.245	0.621
Illness duration, mean ± SD, m	5.37 ± 3.46	5.28 ± 3.38	0.044	0.834
Age at onset, mean ± SD, y	24.70 ± 5.28	24.40 ± 5.26	0.214	0.644
Gender			1.531	0.222
Male, *N* (%)	171 (39.0%)	38 (46.3%)		
Female, *N* (%)	267 (61.0%)	44 (53.7%)		
Education level			1.804	0.187
Middle school, *N* (%)	233 (53.2%)	37 (45.1%)		
College degree or above, *N* (%)	205 (46.8%)	45 (54.9%)		
Marital status			0.016	0.905
Married, *N* (%)	205 (46.8%)	39 (47.6%)		
Unmarried, *N* (%)	233 (53.2%)	43 (52.4%)		
BMI, mean ± SD, kg/m^2^	24.58 ± 2.11	24.74 ± 2.18	0.370	0.543
**Scale assessment**				
HAMD, mean ± SD	31.22 ± 2.68	31.32 ± 2.81	0.083	0.774
HAMA, median [IQR]	20 (18–22)	27 (26–28)	−14.439	<0.001^***^
**Biological indicators**				
TSH, median [IQR], mIU/L	5.98 (4.96–7.35)	7.33 (6.26–9.41)	−6.364	<0.001^***^
TgAb, median [IQR], IU/L	22.14 (14.35–73.50)	23.21 (17.19–119.05)	−1.495	0.135
TPOAb, median [IQR], IU/L	19.78 (12.44–47.66)	22.35 (13.20–93.34)	−1.273	0.203
FT3, median [IQR], pmol/L	5.07 (4.39–5.54)	5.12 (4.33–5.47)	−0.405	0.686
FT4, median [IQR], pmol/L	16.33 (14.21–18.71)	16.81 (14.70–18.94)	−1.430	0.153

*SD, standard deviation; IQR, interquartile range; FEDN MDD, first episode and drug naïve major depressive disorder; A-MDD, anxious major depressive disorder; NA-MDD, non-anxious major depressive disorder; BMI, body mass index; HAMD, Hamilton Depression Rating Scale; TSH, thyroid stimulating hormone; TgAb, anti-thyroglobulin; TPOAb, thyroid peroxidases antibody; FT3, free triiodothyronine; FT4, free thyroxine;*

*** *p < 0.001*.

### Factors Associated With A-MDD in Young FEDN MDD With SCH

**Figure 1:**
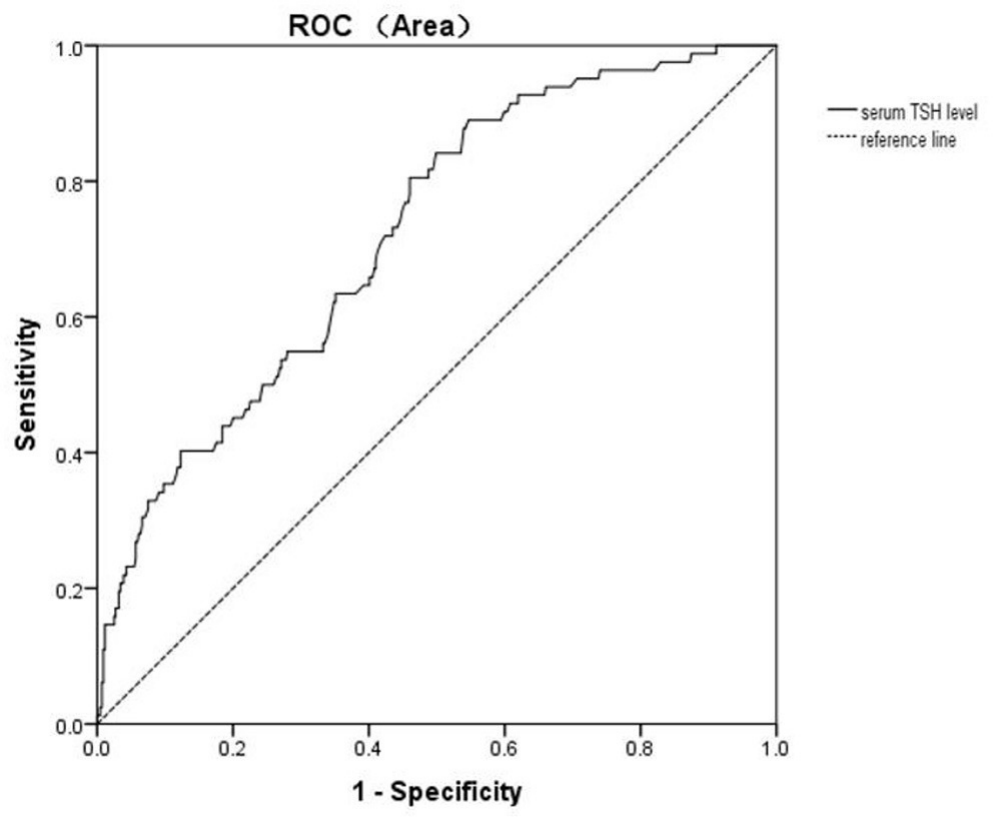
Diagnostic accuracy of serum TSH level for A-MDD in young FEDN MDD patients with SCH. The area under the ROC curve is 0.721 on serum TSH level.

Univariate binary logistic regression showed that serum TSH levels (OR = 1.515; 95% CI: 1.336–1.717) were associated with A-MDD. Multivariate binary logistic regression showed that serum TSH levels (OR = 1.602; 95%CI: 1.388–1.849) were associated with A-MDD (Table 2). The performance of serum TSH level was evaluated according to the ROC curve. The area under the ROC curve of serum TSH level was 0.721 (*p* < 0.001; 95%CI: 0.664–0.778), and its optimal cut-off value was ≥6.17 mIU/L (Figure 1). The sensitivity and specificity of the cut-off point for serum TSH levels were 0.805 (95% CI: 0.703–0.884) and 0.539 (95% CI: 0.491–0.586), respectively.

**Table 2:** Multivariate binary logistic regression for factors associated with anxiety symptom in young FEDN MDD patients with SCH.

**Variable**	**β**	**Odds ratio**	**95% Confidence interval**		***P***
			**Lower**	**Upper**	
Age	−0.330	0.719	0.286	1.810	0.484
Illness duration	0.009	1.009	0.910	1.118	0.868
Age at onset	0.277	1.319	0.523	3.327	0.557
Gender	−0.218	0.804	0.480	1.347	0.407
Education level	0.258	1.294	0.765	2.190	0.336
Marital status	0.494	1.640	0.739	3.636	0.224
BMI	0.055	1.057	0.941	1.186	0.349
HAMD	0.049	1.050	0.956	1.152	0.307
TSH	0.471	1.602	1.388	1.849	<0.001^***^
TgAb	−0.001	0.999	0.998	1.000	0.121
TPOAb	0.000	1.000	0.999	1.001	1.000
FT3	−0.054	0.948	0.663	1.354	0.768
FT4	0.041	1.042	0.958	1.132	0.338

*** *p <0.001*.

## Discussion

To the best of our knowledge, this is the first study to investigate the association between SCH and anxiety symptom in young FEDN MDD patients. Our study revealed that the A-MDD rate among young FEDN MDD patients with SCH was 15.8%. We did not find significant socio-demographical differences between A-MDD and NA-MDD. As for blood samples, serum TSH level in A-MDD was higher than in NA-MDD. Further logistic regression analysis showed that higher serum TSH level was associated with A-MDD, suggesting it a predictive biomarker for A-MDD patients. The cut-off serum TSH level for distinguishing A-MDD was 6.17 mIU/L.

According to previous studies in Germany and the United States, A-MDD accounted for approximately half of MDD patients (33, 34). However, in our study, the prevalence of A-MDD among young FEDN MDD patients with SCH was 15.8%, appearing an obvious decrease. Although the lifetime prevalence of depression is significantly higher in patients with SCH (35), a cross-sectional study with a large sample size suggested that SCH is negatively associated with anxiety disorder (23), which may be a leading reason for the decrease found in our study. It is also of note that patients in our study were recruited from outpatient clinics, with illness duration of no more than 24 months and at the first stage of MDD, which may imply less and milder comorbidity, thus leading to the significant decrease of A-MDD rate. Moreover, Belov and Pshuk found that the severity of anxiety would increase with aging (36), with Lin and colleagues also suggesting that patients with A-MDD were likely to be older (8). Since patients in our study were of a younger population under the age of 36, this further suggests that age was a potential influencing factor contributing to the lower prevalence of A-MDD in this study. In addition, previous studies assessed anxiety using scale systems such as HAM-D anxiety-somatization factor (ASF), which may inflate false positive diagnosis of A-MDD (32). In contrast, our study employed the HAMA, recognized as the gold standard of anxiety severity assessment (32), indicating greater credibility.

One of the critical findings in our current study is that high serum TSH level is associated with A-MDD in young FEDN MDD with SCH. TH play essential roles in the adult brain, and depression and anxiety are commonly observed in hypothyroidism patients (11), demonstrating the importance of TH in regulating mood. The HPT axis works via negative feedback regulation. Absolute or relative TH deficit may lead to the secretion of thyrotropin-releasing hormone (TRH) and TSH. Though SCH patients displayed a normal range of serum FT3 and FT4 levels, the elevated serum TSH level was a potential indication of relative TH deficit, which may cause by decreased receptor sensitivity. Our study is consistent with many previous studies. Andrade and colleagues found a higher risk of anxiety and depression in SCH, and recommended TSH screening for symptom assessment (37). A community-based population study in Brazil suggested a positive relationship between high TSH levels and anxiety and depression in patients with T4 treatment (38). Teixeira Pde et al. reported positive relationships between the severity of depression, anxiety and TSH level (39). An Indian study revealed that after receiving treatment, the severity of anxious and depressive symptoms was decreased in depression patients with SCH. Besides, they also observed a serum TSH level decrease (28), suggesting parallel relationship with anxiety severity in depressive patients with SCH. However, a systematic review reported a negative relationship between anxiety severity and TSH in people without thyroid diseases when looking at patients in two large sample studies (18, 38, 40). Ittermann et al. observed an association between hypothyroidism and anxiety, depression, but no significant correlation between the symptoms and serum TSH level (19). Several reasons may account for the discrepancy. Firstly, some results were derived from secondary analysis and failed to adjust these for relevant confounders (18). Secondly, participants in our study are SCH, whose HPT axis may function abnormally, rendering different TSH secretion in SCH with other population groups. Thirdly, patients recruited in our study received no previous antidepressant treatment, which may influence TSH level (15, 28), while the previous studies may have had different inclusion criteria. Fourthly, participants in various studies included widely varying age groups. The endocrine system is volatile during climacteric. Furthermore, endogenous depression or menopause-relative psychological manifestations may affect the results (11). Since our study included young patients under the age of 36, it is possible to reach different conclusion than with studies comprising of older age groups.

In our study, serum FT3, FT4, TgAb, and TPOAb levels have no significant differences between A-MDD and NA-MDD. Hitherto, no consensus has been reached among previous studies. Wu et al. suggested FT4 as an independent biomarker related to anxiety and depression in AID patients (41). An animal experiment with male mice demonstrated T3 and T4 supplement effective anxiety regulators (14), while Yu et al. observed decreased anxiety and depression in mice with lower FT3 and FT4 levels (15). A study targeting Chinese females saw no significant correlation between hair T3 or T4 levels and the HAMA and HAMD scores, as well as hair TH level changes in different episodes of depression (42). Yang and colleagues' research targeted a similar population, revealing a negative correlation of hair T3 levels with anxiety and depression severity in first-episode patients with major depressive disorder (F-MDD) (43). A recent study found FT4 values within the normal range were associated with the severity of depression and anxiety (12). Though untreated diagnosed hypothyroidism was positively related to depressive symptoms and anxiety, it appeared no significant link existed between these two symptomologies and TPOAb (19). The possible reasons for these variable conclusions may be the inconsistent study design in our and previous studies. Samples from different body parts or collecting time may lead to different results. Subjects within different disease episodes can also partly explain the discrepancy among the studies (25, 42).

No significant demographical differences were found between A-MDD and NA-MDD in this study. Previous studies have reached variable conclusions. A cross-sectional study in India suggested no significant gender difference existed in HAMA score in hypothyroidism patients (11). A Korean review indicated that patients with anxious depression are more likely to be female gender, non-single and less educated (7). Another study conducted in the USA revealed a higher risk of anxious depression among those who were unemployed, with less education and once married or married (34). A German randomized controlled trial (RCT) listed older age, lower education levels and longer duration of the current episode as risk factors of anxious depression (33). Belov and Pshuk postulated that the severity of anxiety is associated with older age and female gender in depressive disorder (36), though this study included a mixed population with bipolar affective disorder (current episode of depression), depressive episode and recurrent depressive disorder. However, no prior studies have been conducted in young FEDN MDD patients with SCH to explore demographical features between A-MDD and NA-MDD.

To the best of our knowledge, our study is the first to explore associations between SCH and anxiety symptom in young FENN MDD patients. Hormones in the HPT axis have long been considered significant regulators of anxiety and depression (18, 38, 40). As the endocrine system is easily influenced by age, race, endocrine disease states, episodes of psychiatric disorders and medical treatment, our study focused on young FEDN MDD with SCH in the Chinese Han population to minimize these disturbances. Therefore, our results might be more accurate in clinical practice toward this particular group. It may contribute to predicting the severity of anxiety symptom in young FEDN MDD with SCH patients, thus appropriate evidence-based clinical prevention or intervention can be imposed. Compared with previous studies, this study employed HAMA, which is considered as the gold standard for anxiety assessment, rather than ASF to distinguish A-MDD patients, indicating a more concrete result. Moreover, given both SCH (44) and MDD (4, 6, 45) are more frequently diagnosed in young adults, our study appears to have more profound practicality.

Several limitations should be noted in our study. Firstly, as a cross-sectional study, its effectiveness in explaining the causal relationship between SCH and anxiety symptom in young FEDN MDD patients is limited. Further longitudinal studies are indispensable to investigate their causal relationship. Secondly, the medication history was obtained by interviewing patients and their family members instead of medical records. Thirdly, several confounding factors critical to the study, such as family income, diet and serum TSH level before the onset of MDD, which should be remedied in future studies by doing a prospective study. Fourthly, all MDD patients were recruited from the outpatient department of a regional general hospital. Therefore, our findings should be extended with caution to inpatients, community patients, and outpatients from other regions or racial groups. Fifthly, in this study, the recruitment of FEDN patients was used to avoid potential influence by medication and disease episodes. However, this led to the limitation of its extension to the entire MDD patient populations. Future research should involve MDD patients in different phases. Moreover, although all patients received a second diagnosis in the following 3–6 months and only patients with consistent MDD diagnosis were enrolled, it is possible to include diseases other than MDD. For instance, bipolar disorder and schizophrenia can manifest as depression in certain disease stages. As a sixth consideration, pregnant or lactating women were excluded from this study, given their drastic endocrine differences. Therefore, future research targeting this group is certainly needed. Seventhly, we only collected a blood sample one time. Repeated thyroid hormone tests within at least 3 months apart were recommended for a determined SCH diagnosis (46). Future study should impose repetitive examinations of TSH, T4 and T3 levels for a more accurate assessment of SCH. Total thyroxine (TT4) and total triiodothyronine (TT3) should also be included as the complete profile of thyroid hormones. Eighthly, our research results should be considered preliminary due to a lack of healthy control group, expecting future studies to confirm and replicate. Finally, serum TSH level was recommended as a potential predictive biomarker for A-MDD and a cut-off value of serum TSH level for distinguishing A-MDD was given. However, the critical value was low in specificity. Further studies are needed for a more accurate critical value with high specificity and sensitivity.

## Conclusion

In conclusion, this study suggested that the A-MDD rate among young FEDN MDD patients with SCH was 15.8%. There is no significant age, gender, age at onset, illness duration, marital status, education level and BMI difference between A-MDD and NA-MDD among young FEDN MDD patients with SCH. Serum TSH level may serve as a potential biomarker for A-MDD, which is equivalent to MDD with moderate to severe anxiety symptoms, in young FEDN MDD patients with SCH. While serum TSH level was over 6.17 mIU/L, patients were more likely to experience moderate to severe anxiety symptoms. We recommend that young FEDN MDD patients with SCH have their serum TSH levels checked to better assess the severity of anxiety symptom.

## Data Availability Statement

The raw data supporting the conclusions of this article will be made available by the authors, without undue reservation.

## Ethics Statement

The studies involving human participants were reviewed and approved by the Medical Ethics Committee in First Hospital of Shanxi Medical University. The patients/participants provided their written informed consent to participate in this study.

## Author Contributions

RY: conceptualization, methodology, software, investigation, formal analysis, and writing—original draft. ZL, XZ, XLy, GY, XLu, GZ, and CL: data curation, visualization, and investigation. YY, YW, and RP: resources, supervision, and software. YZ, HW, and SW: software and validation. PG and HEW: visualization and writing—review and editing. XD and XZ: conceptualization, funding acquisition, resources, supervision, and writing—review and editing. All authors contributed to the article and approved the submitted version.

## Funding

This work was supported by the Jiangsu Province High-level Health Talents Six-one Projects (LGY2020042), Jiangsu Province 333 Project scientific research project (BRA2020120), Key Diagnosis and treatment Program of Suzhou (LCZX202016), and the Suzhou clinical Medical Center for mood disorders (Szlcyxzx202109).

## Conflict of Interest

The authors declare that the research was conducted in the absence of any commercial or financial relationships that could be construed as a potential conflict of interest.

## Publisher's Note

All claims expressed in this article are solely those of the authors and do not necessarily represent those of their affiliated organizations, or those of the publisher, the editors and the reviewers. Any product that may be evaluated in this article, or claim that may be made by its manufacturer, is not guaranteed or endorsed by the publisher.
